# Health care seeking among detained undocumented migrants: a cross-sectional study

**DOI:** 10.1186/1471-2458-11-190

**Published:** 2011-03-28

**Authors:** Tina Dorn, Manon Ceelen, Ming-Jan Tang, Joyce L Browne, Koos JC de Keijzer, Marcel CA Buster, Kees Das

**Affiliations:** 1 Public Health Service, Department of Epidemiology, Documentation and Health Promotion, P.O. Box 2200, 1000 CE Amsterdam, The Netherlands; 2 Public Health Service, Department of Forensic Medicine, P.O. Box 2200, 1000 CE Amsterdam, The Netherlands

## Abstract

**Background:**

As in many European countries, access to care is decreased for undocumented migrants in the Netherlands due to legislation. Studies on the health of undocumented migrants in Europe are scarce and focus on care-seeking migrants. Not much is known on those who do not seek care.

**Methods:**

This cross-sectional study includes both respondents who did and did not seek care, namely undocumented migrants who have been incarcerated in a detention centre while awaiting expulsion to their country of origin. A consecutive sample of all new arrivals was studied. Data were collected through structured interviews and reviews of medical records.

**Results:**

Among the 224 male migrants who arrived at the detention centre between May and July 2008, 173 persons were interviewed. 122 respondents met inclusion criteria. Only half of the undocumented migrants in this study knew how to get access to medical care in the Netherlands if in need. Forty-six percent of respondents reported to have sought medical help during their stay in the Netherlands while having no health insurance (n = 57). Care was sought most frequently for injuries and dental problems. About 25% of these care seekers reported to have been denied care by a health care provider. Asian migrants were significantly less likely to seek care when compared to other ethnic groups, independent from age, chronic health problems and length of stay in the Netherlands.

**Conclusion:**

The study underlines the need for a better education of undocumented patients and providers concerning the opportunities for health care in the Netherlands. Moreover, there is a need to further clarify the reasons for the denial of care to undocumented patients, as well as the barriers to health care as perceived by undocumented migrants.

## Background

Illegal migration is a fact of live in many European countries. In the Netherlands, the number of undocumented migrants was estimated 130,000 in 2005 [1]. This group includes people who entered the country clandestinely, overstayed their visas, violated the terms of their admission, or were rejected as asylum seekers. The access to health care of this group is compromised due to various obstacles. First, in the Netherlands, it is impossible for undocumented migrants to join the national health insurance system, leading to a decreased accessibility of care. Second, migrants may avoid contacting health care providers out of fear of being denounced to immigration authorities [2,3].

Dutch legislation plays a key role in many of the obstacles undocumented migrants face in health care provision. The *Koppelingswet *('linking act') of 1998 intends to discourage illegal residency in the Netherlands by linking the usage of public services, such as welfare, housing benefits, student grants and health insurance, to legal status. Health care providers, however, have the legal and moral duty to provide medical care to everyone, also to uninsured and/or undocumented residents. Therefore, funding was made available for the provision of 'medically necessary care' to these groups of patients. The term 'medically necessary care', however, is not well defined, causing much discussion among health care professionals on what kind of care can be provided [4]. Moreover, bureaucratic procedures necessary to obtain substitution of costs for the treatment of undocumented migrants is burdensome to health care providers. Past research in the Netherlands demonstrated that not only patients, but also providers can be ignorant about these opportunities for reimbursement of costs [2,5,6]. Altogether, these factors endanger undocumented migrants' access to health care.

Research on documented migrants in the Netherlands often focuses on the most prominent ethnic minority populations, that is, Turkish and Moroccan labour migrants. From this research, it is known that both groups are well represented in general practice, whereas outpatient and mental health care are underutilized when compared to ethnic Dutch [7,8]. Not surprisingly, language ability appears to have a central role in the use of health care services by these groups [9]. With respect to mental health, it is assumed that the life transitions involved in migration and factors such as acculturation and sociodemographic position affect psychological functioning and mental health. Nevertheless, some migrant groups can appear to be in good health when compared to the native population [10] and demonstrate a similar health seeking behaviour as the Dutch natives when it comes to mental health care [11].

A recent review of the international literature on primary care use of migrant groups living in 7 different countries [12] failed to establish an overall consistent pattern of differences between migrant and non-migrant groups. In the US, generally a lower primary care use of migrant groups is reported than in European studies, which is probably attributable to characteristics of the US health care system such as the lack of universal health insurance. Furthermore, studies adjusting for health status less frequently reported differences between migrant and non-migrant groups, as poorer health and lower socioeconomic status seem to account for the higher use of health care among migrants [12,13].

Documented and undocumented migrants share the same motives for migration: they leave their countries due to poverty and political instability. Legal migrants, however, are given the opportunity to build up a life in the host country and enjoy access to health care and social security, undocumented migrants are threatened by expulsion to their home country. To conclude, their socioeconomic position can be considered worse than that of documented migrants.

Not surprisingly, undocumented migrants are underrepresented in the research literature on health care utilization, especially in European countries. One of the underlying reasons probably is that research on the health of undocumented residents is difficult to perform since they belong to a hidden population. Due to mistrust in official institutions, undocumented migrants are less likely to respond to formal surveys. Not surprisingly, existing research in Europe concentrated on those who consulted health care services, providing an indication of the medical consumption and type of health problems of undocumented migrants who seek care, sometimes with contradicting results [14-22].

Unlike previous studies, the current study includes both respondents who did and did not seek care, namely undocumented migrants who have been incarcerated in a detention centre while awaiting expulsion to their country of origin. This setting provides the opportunity to gather basic epidemiological data on the health and health care utilization of both care and non care-seeking undocumented migrants.

The research questions posed are:

1. Which proportion of the respondents knows how to get access to medical care in the Netherlands?

2. Which proportion of the respondents has sought medical care in the Netherlands ('care seekers')?

3. In case of health problems, what type of health care provider did they consult and which health problems did they present?

4. Which proportion of the respondents reports to have been denied care by a health care provider during their stay in the Netherlands?

5. What are determinants of care-seeking in undocumented migrants?

## Methods

### Study design

A cross-sectional study was performed among undocumented migrants who were incarcerated on one of the two detention platforms located in Zaandam, a city 15 km north of Amsterdam.

### Setting

Zaandam's platforms can house a maximum of 576 male detainees at two men to a cell [23]. Their offence is being in the country without the necessary documents (e.g. irregularly crossing the border, using false documents, irregular stay, breaching or overstaying their conditions of stay). The main difference with criminal incarceration is that the detention is not the result of a trial, but based on the reasoning that persons awaiting their expulsion while not in custody run a risk of their absconding [24,25].

### Participants

The study aimed to include all persons who newly arrived at the detention centre between May 15 and July 17, 2008 (consecutive sample). For this purpose, the detention centre's administration department provided the research team on a regular basis with a list of names and cell numbers of all new arrivals. These persons were then approached by the interviewer. Before the interview, the interviewer introduced herself as an employee of the Amsterdam Public Health Service and provided information on the content and duration of the interview. It was emphasized that the interviewer was not related to the Ministry of Justice and its staff, and that information provided during the interview would not be shared with others. Hereafter, since the interview was on a voluntary basis, oral informed consent for participation was obtained in Dutch, English or French by the interviewer. If the respondent was unable to communicate in one of these languages, a telephone interpretation service was contacted for assistance. During the interviews, only the interviewer and the respondent were present in order to guarantee confidentiality. The first interviews took place on May 28, the last interviews on July 17, 2008. Interviews were carried out one to two times a week, depending on the number of new arrivals. If the participant was temporarily unavailable (e.g. participant was on transport to embassy or receiving visitors), a new attempt was made on the following occasion. Since the study protocol was in accordance with the Dutch Medical Research involving Human Subjects Act (WMO), it was not subjected to an ethics committee.

### Measurement

As a conceptual model, we used an adaption of Andersens' behavioral model of health services use as a framework to study health care seeking [26]. The model is based on three determinants of health care use, which we will elaborate below. The first determinant is 'need' which refers to ill-health, since it initiates the decision to seek care. The second determinant concerns so-called 'enabling factors' which reflect the economic means and human factors (e.g. knowledge, education). The third determinant compromises 'pre-disposing factors', including beliefs and attitudes regarding health and use of specific services. In migrants, these attitudes are influenced by processes of acculturation. Several studies on the relation between acculturation and service use suggest that increased adaption to the host culture is associated with higher service use [9].

In this study, 'need' was operationalized as suffering from chronic disease. Second, higher age was considered as a factor influencing ill-health and thus the 'need' determinant. As 'enabling factor', we measured knowledge of where to turn in case of health problems. Next to this, region of origin was included. Region of origin as an predisposing factor was considered because it can be assumed that migrants who belong to one of the larger migrant groups in the Netherlands are more likely to find their way in the Dutch health care system as these migrants are part of a larger group which is well acquainted with the host country, in contrast to members belonging to smaller, marginal migrant groups who lack sources of informal support and advice in the case of sickness.

Furthermore, care seekers were defined as persons seeking care with officially registered care providers while not having health insurance at that point in time. This definition excludes care seeking with informal care providers such as traditional healers, as the study concentrates on health care seeking within the formal health care system.

For the purpose of the study, two sources of information were used. First, a structured questionnaire was developed (see Additional file 1). The questionnaire was pre-tested with respondents incarcerated in Zaandam. As a consequence of the pre-test, the questionnaire was further simplified and the number of questions reduced. The questionnaire was available in Dutch, English and French. If the respondent was unable to communicate in one of these languages, assistance from a telephone interpretation service was sought. In addition, during an intake procedure shortly after arrival at the detention centre, the health care needs of every detainee were documented by a nurse by posing a standard set of questions. These medical records were assessed to get insight in the medical history and current complaints of the respondents.

### Analyses

Respondents who reported to have spent less than three months in freedom in the Netherlands were excluded from the analyses, since the period of stay was considered too short to draw conclusions. Next to this, respondents who reported to have been insured for medical expenses during their whole stay in the Netherlands were excluded. This is the case in former asylum seekers who have been transferred to the detention centre after refusal of their application.

Descriptive statistics (frequency distributions and means) were used to describe the basic features of the data. Differences between care seeking and non care seeking respondents were tested using t-tests and chi-square tests. Multivariate logistic regression was applied to study determinants of care seeking in the study population. In the analysis, we included age, chronic health problems, duration of stay in the Netherlands and country of origin as determinants. All analyses were carried out using SPSS 17 for Windows.

## Results

Among the 224 migrants who arrived at the detention centre during the study period, 173 persons were interviewed (77%). On average, the interview took place nine days after arrival (SD = 6). Non-participation in the study was predominantly due to respondents being released from custody before the interview could take place (n = 45). Another four potential participants were unwilling to contribute to the interviews. Two other persons could not be interviewed because the situation was not considered to be safe for the interviewer. Respondents who reported to have spent less than three months in freedom in the Netherlands (36 out of 173) were excluded from the analyses, as well as those who reported to have been insured for medical expenses (n = 15), resulting in a sample of 122 respondents. Respondents in the final sample were aged 18 to 57 years (mean 33.2; SD 9.1) and had lived in the Netherlands for 8.2 years on average (SD = 6.3; median = 7; Q_3_-Q_1 _= 6). 34% of the respondents originated from sub-Saharan Africa, 26% from North Africa, 17% from Asia, 13% from the Middle East, 9% from Latin America and 1% from Eastern Europe (table 1). As visible in the univariate analysis, the percentage of care seekers was lowest in Asian respondents. Among the 21 Asians interviewed, 11 originated from India (1 care seeker), 7 from China (1 care seeker), 2 from Nepal and 1 from Indonesia.

**Table 1:** Characteristics of respondents according to care seeking

	Total sample n = 122	Care seekers n = 57	Non-care seekers n = 65	p-value †
Mean age (SD)	33.2 (9.1)	34.9 (9.7)	31.7 (8.3)	0.06

Mean length of stay in years (SD)	8.2 (6.3)	9.8 (7.2)	6.8 (5.1)	0.01*

Region of origin^#^				

Sub-Saharan Africa (%)	41 (33.6)	22 (38.6)	19 (29.2)	0.28

North Africa (%)	32 (26.2)	19 (33.3)	13 (20.2)	0.09

Asia (%)	21 (17.2)	2 (3.5)	19 (29.2)	0.000***

Middle East (%)	16 (13.1)	9 (15.7)	7 (10.7)	0.41

Latin America (%)	11 (9.0)	5 (8.7)	6 (9.2)	0.93

Eastern Europe (%)	1 (0.8)	0	1 (1.5)	-

*p < 0.05, ***p < 0.001, † based on two-sided analysis; independent samples t-test for continuous data, chi-square test for discrete data;

^# ^Sub-Saharan Africa: Angola, Burundi, Congo, Eritrea, Ethiopia, Ghana, Ivory Coast, Cameroon, Liberia, Mauretania, Nigeria, Ruanda, Sierra Leone, Somalia, Sudan, Chad; North Africa: Algeria, Egypt, Morocco, Tunisia; Asia: China, India, Indonesia, Nepal; Middle East: Iran, Iraq, Israel, Lebanon, Pakistan, Turkey; Latin America: Colombia, Cuba, Guyana, Suriname; Eastern Europe: Georgia.

The first research question concerned the proportion of respondents who knew how to get access to medical care in the Netherlands. Forty-eight percent of the respondents reported that they knew how to find medical help if needed (n = 59). Thirteen percent indicated that they did not know how to get access to medical care (n = 16). Fifty-seven respondents stated that they had sought medical help during their stay in the Netherlands although they had been uninsured ('care seekers'). In table 2, it is illustrated which care providers have been consulted according to the respondents and the most prevalent health problems for which the respondents consulted a health care provider. Fourteen out of 57 care seekers reported to have been refused treatment at least once during their stay in the Netherlands (24.6%). In most cases, this concerned hospitals (n = 9). Respondents who were refused at hospitals reported the following health problems: injuries (n = 2), mental health problems (n = 2), trouble breathing (n = 1), haemorrhoids (n = 1), tooth ache (n = 1), hepatitis (n = 1) and eye problems (n = 1). One of the respondents was denied care by a dentist when presenting with tooth ache. The other respondents were refused by general practitioners with nose problems (n = 1), dizziness/headache (n = 1), kidney problems (n = 1) and skin problems (n = 1).

**Table 2:** Care seekers (n = 57): Type of provider consulted, health problems for which care was sought and number of care seekers who were denied care

	n	%
Type of provider		

Hospital	20	32.3

General practitioner	18	29.0

Clinic for the uninsured and/or homeless	17	27.4

Dentist	7	11.3

Sum of providers mentioned	62	

Type of health problem presented		

Injury	26	18.6

Dental problems	20	14.3

Gastrointestinal problems	17	12.1

Infectious diseases	14	10.0

Musculoskeletal problems	14	10.0

Respiratory problems	13	9.2

Chronic diseases	9	6.4

Cardiovascular problems	7	5.0

Mental problems	4	2.9

Eye problems	4	2.9

Ear problems	4	2.9

Skin problems	4	2.9

Vaccinations	4	2.9

Sum of health problems	140	

Number of care seekers who were denied care	14	24.5

Next to the interviews, the medical files of the respondents were checked for chronic diseases (coded according to the classification by Hoeymans et al.) [27], past suicidal thoughts or attempts, complaints and medication use at the moment of arrival at the detention centre. Information was available for 95 of the 122 respondents who met inclusion criteria for the interviews (78%; table 3).

**Table 3:** Information collected during the medical intake procedure by the nurse (n = 95)

	Total sample n = 95	Care seekers n = 44	Non-care seekers n = 51	p-value†
Current medication use (%)	19 (20.0)	14 (31.8)	5 (9.8)	0.007*

Asks attention for current health problem (%)	32 (23.2)	18 (40.9)	14 (27.4)	0.17

Past suicidal thoughts and/or attempts (%)	10 (11.4)	4 (9.1)	6 (11.7)	0.67

History of one or more chronic health problems (%)	14 (14.7)	8 (18.1)	6 (11.7)	0.38

Number of chronic health problems	n = 16	n = 10	n = 7	

Chronic back/neck pain	3	1	2	

Hearing problem	2	1	1	

HIV/AIDS	2	2	0	

COPD	2	2	0	

Vision problem	1	1	0	

Heart disease	1	0	1	

Chronic alcohol abuse	1	0	1	

Depression	1	1	0	

Eczema	1	0	1	

Tuberculosis	1	1	1	

CVA	1	1	0	

**p < 0.01, † based on two-sided analysis; chi-square test

Twenty percent of these 95 individuals reported that they were currently using medications, with care-seekers using medications significantly more often than non-care seekers (p = 0.007). Other differences between the two groups did not reach statistical significance (table 3). Finally, we tested which factors were related to health care seeking, combining information stemming from the interviews and the medical files. The results of the logistic regression demonstrated that when all determinants were considered simultaneously in one model, neither age, nor chronic health problems, nor duration of stay in the Netherlands significantly influenced the odds of care seeking, while respondents originating from an Asian country were significantly less likely to seek care (table 4).

**Table 4:** Determinants of health care seeking in undocumented migrants (multivariate logistic regression)

	Odds ratio	95% CI	p-value
Chronic health problems (yes = 1, no = 0)	1.37	0.39-4.86	0.62

Age (years)	1.01	0.96-1.07	0.70

Region of origin (Asia = 1, other = 0)	0.08	0.01-0.65	0.02*

Duration of stay (years)	1.06	0.98-1.15	0.16

*p < 0.05; Model chi-square (likelihood ratio chi-square) = 17.44, df = 4, p = 0.02; Nagelkerke Pseudo-R-square = 0.224

## Discussion

The data presented here provide a glimpse into the background, types of medical complaints and health care seeking behaviour of undocumented male migrants in the Netherlands. Since respondents were interviewed in a detention setting, in contrast to previous studies, information on undocumented migrants who did not seek care could be collected as well.

Univariate analyses demonstrated that undocumented migrants differed from care-seeking migrants with respect to the time being in the country, but not with respect to age and chronic health problems. Moreover, non-care seekers more often originated from Asian countries when compared to those who had sought care. Forty-six percent actually consulted a health care provider during their stay in the Netherlands. If care was sought, hospitals were most often consulted, followed by general practitioners and clinics for underserved populations such as the homeless and uninsured. Injuries and dental problems were the leading reasons for seeking care. About 25% of the care-seeking undocumented migrants reported to have been refused treatment by a care provider while in the Netherlands. According to medical files, 15% of both care and non-care seekers suffered from chronic health problems, 11% reported past suicidal thoughts and 20% were using medication.

A strength of the study is that the participation rate for the interviews amounted to 77%. Non-participation was predominantly due to external factors which were largely unrelated to the study outcomes, meaning that the studied sample can be considered representative for the detention centre's population. Another major strength is the inclusion of both care and non-care seeking undocumented migrants. To our knowledge, there are no studies so far systematically comparing these two groups. Usually, reference groups for undocumented migrants consist of regular Dutch patients e.g. in primary care or emergency rooms. The current study is unique in this respect, since it makes use of a particular environment in which both care and non-care seeking individuals can be found.

Performing research in this setting has advantages, but also its limitations. First, the small sample size can be considered as a weakness of the study, as this decreases the power of the study and increases the susceptibility to sampling error. A related issue concerns the representativeness of the study sample for the undocumented migrant population in the Netherlands. It cannot be assumed that our sample is a random selection of male undocumented migrants in general since it was performed among those who caught the attention of the police, leading to identification and subsequent incarceration. This could mean that the results of our study are not representative for the population of undocumented aliens in the Netherlands, with delinquent individuals being overrepresented. Nevertheless, it has to be taken into account that the Netherlands gradually introduced harsher immigration measures in the last decade. As a result, an increasing number of undocumented migrants are held in special detention units while awaiting expulsion to their country of origin. It is important to keep in mind that the large majority of individuals enclosed in our study population has not been incarcerated because they are suspects of crime or proven offenders, but for residing illegally in the Netherlands [24].

It is hard to judge how representative the population encountered in the detention centre is for the population of undocumented migrants in the Netherlands, as the size and characteristics of this hidden population can only be estimated. According to the most recent estimates, approximately 130,000 undocumented migrants live in the Netherlands [1]. Approximately 70% of these migrants come from non-European countries, the other 30% from European countries. In our sample, however, almost all respondents originated from non-European countries, meaning that our conclusions most probably apply to this proportion of undocumented migrants. Other studies on illegal migration in the Netherlands indicated that 75-85% of undocumented migrants are male and that approximately three quarters is aged 20-40 years [28,29]. This age distribution is comparable to the one found in our study sample.

Another point of discussion concerns whether the findings of our study also might apply to asylum seekers and refugees who newly arrived in the Netherlands. These groups face similar problems, that is, the initial lack of knowledge on the Dutch health care system, language problems and difficulties arising in the communication with health care providers who have a different cultural background than the patient. These barriers to health care provision and access have been extensively described in the literature [30,31]. Undocumented migrants also suffer from these barriers, but in contrast to asylum seekers and documented migrants, they cannot access the Dutch health insurance system due to legislative measures put in place. Instead, they are expected to pay for their own health expenses. In addition, in contrast to other migrant groups, undocumented migrants run the risk of being identified as illegal residents when contacting health care providers, creating a different set of problems concerning health care access.

In addition, our study results may have been influenced by information bias. In order to establish the medical history of the respondents, the medical files of the participants were used as a source of information. As a standard procedure, nurses see those who arrive at the detention centre for an intake procedure. The aim of this procedure is to gain information on the health needs of detainees. As with any research based on medical files, the absence of written information does not necessarily imply the absence of a health problem. It cannot be excluded that certain conditions are underdiagnosed, especially in those who have not sought care in the past. Information bias thus could decrease the differences observed between care seekers and non-care seekers regarding the prevalence of chronic health problems. Furthermore, detainees with stigmatizing conditions such as HIV, other sexually transmitted diseases or mental health problems might have underreported these problems during the interview with the nurse. Mental health problems in particular can be difficult to assess due to different cultural backgrounds of patients and care providers [32]. It can be argued, however, that this form of underreporting and the resulting underdiagnosis would be equally present in both care and non-care seekers and therefore should not affect differences observed between the two groups. Next to this, differences in chronic health problems between regular patients and undocumented migrants could have been underestimated. According to national statistics based on the same definition of chronic diseases, about 24% of the male regular patients seen in primary care settings in the Netherlands suffers from chronic diseases [27]. In our study, this percentage is much lower, approximately 15%. Since it is unclear if there was underdiagnosis of chronic conditions in the current study, it cannot be determined for sure if the undocumented migrants studied here can be considered healthier than regular patients in the Netherlands.

Finally, it should be noted that the used questionnaire was conceptualized in a rather broad manner, addressing several aspects of care seeking within a short interview performed in a quite restrictive setting. One might criticize that certain important aspects such as the denial of care should have been explored more in-depth. For example, the study cannot answer the question whether a doctor, nurse or administrator refused care to the patient and whether the patients were able to overcome access barriers by contacting another provider. Further research on this issue should therefore collect information on these aspects.

## Conclusions

After having described the strengths and limitations of this study, implications for researchers and policy makers should be discussed. This study demonstrates that less than half of the study sample utilizes medical care in the Netherlands. Although our study sample is relatively small, it gives a rough indication of the proportion of the population which remained out of sight in earlier studies. Moreover, our study demonstrated that Asian migrants in particular were significantly less likely to seek care in the Netherlands when compared to undocumented migrants from other regions of origin. Although there is a considerable body of literature on the traditional health care practices of Chinese migrants [33], it should be noted that among the Asian detainees we interviewed, the majority originated from India and not from China. This fact considerably limits the value of this explanation for our sample. A better explanation for the finding that Asian migrants in this study less likely sought care than other groups, might be that this group of migrants faces language problems. This is supported by the fact that almost half of all interviews with Asian detainees have been performed with the help of an interpreter, in contrast to only one tenth of the interviews held with migrants from other regions. Moreover, it could be speculated that Asian migrants are more marginalized because they belong to a migrant group which is small in size in the Netherlands. Therefore, these migrants might lack the informal support and advice of other compatriots who already know how to access the Dutch health care system.

Another major result concerns the fact that only half of the undocumented migrants in this study knew how to get access to medical care in the Netherlands if in need. This finding reflects a substantial lack of knowledge on treatment opportunities in the host country which could be improved by a better education of undocumented patients and health care personnel. Since current public policies are directed at discouraging illegal migration, the education of undocumented patients about their rights and opportunities for treatment mainly is in the hands of non-governmental organizations (NGOs). Our research demonstrated that undocumented migrants often consult clinics run by NGOs. A recent cross-national study included 835 undocumented migrants in 7 European countries who consulted free clinics [34]. In this study, one person in ten reported refusal of treatment by health care professionals. In our sample, this number amounted to 25% of those who had sought care in the Netherlands. In contrast to documented migrants who are obliged to participate in national health insurance schemes by law, the rate of care refusal according to this study in undocumented migrants is rather high. In most cases, respondents reported to have been rejected by hospitals. This matches the results of earlier studies carried out at Dutch emergency rooms. Consistent with studies carried out in Denmark and Spain [35,36], Dutch health professionals indicated that undocumented migrants more often presented conditions and complaints which better can be presented elsewhere in the health care system [2]. Also, undocumented migrants more often presented with dental problems at emergency rooms instead of a dentist's office [37]. Partly, this can be attributed to a lack of knowledge on the Dutch health care system on the side of undocumented migrants. It thus cannot be excluded that some of the refusals reported by respondents in this study as a matter of fact were "perceived refusals", fuelled by language problems and miscommunication. As our research did not attempt to clarify the reasons for the denial of care, additional research should shed more light on the underlying processes leading to this phenomenon. In our view, informed by such research, a strategy should be developed to counter the problem. Moreover, it should be explored how health professionals who encounter undocumented migrants in their daily practice can contribute to improved care for this target group. Health care professionals who are faced with undocumented migrants should be alerted by our results as they shed light on the difficulties these patients experience when trying to access the health care system. Good communication skills are essential for health care staff as newcomers who are unfamiliar with the health care system may interpret relatively innocent events as evidence of discrimination or racism if they are not clearly explained [38]. Moreover, staff should be sensitive to cultural aspects in communication. In case of language problems, the help of telephonic interpretation services should be used by health care professionals in order to prevent that negative experiences result in subsequent avoidance of seeking health care.

Of course, explanations for the denial of care must be sought on both the patient and the provider side. Sad enough, there are several case reports concerning the denial of care to undocumented migrants in critical situations [4]. Reasons provided in the literature are a lack of knowledge on reimbursement opportunities and the fact that, in the past, particularly hospitals were not adequately funded to provide care to undocumented migrants. Also, the administrational burden involved when attempting to obtain reimbursement through government funds seems to play a role [4]. Altogether, the refusal of undocumented patients without proper referral and education of the patient are highly unacceptable situations. As of January, 1^st ^2009, new legislation regarding health care for undocumented migrants has been put in place in the Netherlands. In the new system, hospitals can be reimbursed for their income losses as well if the patient is unable or unwilling to pay. General practitioners will receive compensation for 80% of their earning losses and midwives 100%. Although the new regulation has many positive aspects, it is feared that the 80% rule can lead to the refusal of patients by care providers. It therefore is important to critically evaluate the developments in the future [39].

## Competing interests

The authors declare that they have no competing interests.

## Authors' contributions

JB, MJT, MC and TD carried out the interviews. KdK assisted with data collection concerning the medical files. TD performed the statistical analysis. MC, MB, and KD were involved in designing the study, the interpretation of data and helped to draft the manuscript. All authors read and approved the final manuscript.

## Pre-publication history

The pre-publication history for this paper can be accessed here:

http://www.biomedcentral.com/1471-2458/11/190/prepub

## Supplementary Material

Additional file 1 **This file includes the questionnaire used for the interviews performed in this study**. Click here for file
