# Supplementary material for: Health care seeking among detained undocumented migrants: a cross-sectional study
Source: BMC Public Health. 2011 Mar 28;11:190. doi: 10.1186/1471-2458-11-190 (PMC3078097; doi:10.1186/1471-2458-11-190)
Supplement: Additional file 1 — This file includes the questionnaire used for the interviews performed in this study. [file 1471-2458-11-190-S1.PDF]

Interview Detained Undocumented Migrants – English version

Patient number: ☐☐.☐☐.☐☐☐
Name interviewer: ……………
Date: ……………

Language interview: ☐ English ☐ Dutch ☐ Other:……….
Interpreter: ☐ yes ☐ no ☐ not applicable

Question 1. What is your name?

…………………………………………

Question 2. For how long have you been in the Netherlands?

…………………………months
☐ <Unknown>

Question 3. For how long have you been in the Netherlands in freedom?

…………………………months
☐ <Unknown>
<*If less than 3 months or unknown* --> END OF INTERVIEW! *Further with question 20*>

Question 4. For how long have you been in the Netherlands in prison?
…………………………months
☐ <Unknown>

Question 5. Have you lived in a center for asylum seekers in the Netherlands (AZC)?
☐ Yes <*further with question 6a*> ☐ No < *further with question 6b*>
☐ <Unknown>

Question 6a. After your time in the AZC, did you have health insurance in the Netherlands (e.g. 'ziekenfonds')?
☐ Yes, I <u>always</u> had health insurance while in the Netherlands --> END OF INTERVIEW! *Further with question 20*>
☐ Yes, but only from……………to…………….
☐ No
☐ <Unknown>

Question 6b. Did you have health insurance in the Netherlands (e.g. 'ziekenfonds')?
☐ Yes, I <u>always</u> had health insurance while in the Netherlands --> END OF INTERVIEW! *Further with question 20*>

☐ Yes, but only from……………to…………….
☐ No
☐ <Unknown>

Question 7. When you did not have health insurance in the Netherlands, did you know where to find a doctor who would help you?
☐ Yes. I could have gone to……………..
☐ No. I was sick, but I didn't know to which doctor I could go
☐ No. I was <u>never</u> sick and therefore did not have to look for a doctor --> END OF INTERVIEW! *Further with question 20*>
☐ <Unknown>

Question 8. When you did not have insurance in the Netherlands, did you ever go to a doctor?
☐ Yes
☐ No <*Further with question 15!*>
☐ <Unknown>

Question 9. What kind of doctor was this? <several options possible>
☐ General practitioner/family doctor
☐ Specialist
☐ Other, that is <name of organisation>:………………………………
☐ <Unknown>

Question 10. **What kind of health problems did you have at that time?**

……………………………………………..
☐ <Unknown>

Question 11. **Did the doctor help you well?**
☐ Yes
☐ No, because:…………………..
☐ <Unknown>

Question 12. **How many times have you been to that doctor?**
☐ Only once
☐ More than once
☐ <Unknown>

Question 13. **How did you find this doctor?**
☐ Friends/acquaintances
☐ Family
☐ Other, that is:……………………
☐ <Unknown>

Question 14. **Did you have to pay the doctor yourself?**
☐ Yes, approx.……………..Euro
☐ No, I did not have to pay
☐ Other, that is:…………………
☐ <Unknown>

Question 15. **Did it ever happen to you that you did NOT go to a doctor in the Netherlands because you were unable to pay for it?**
☐ Yes explanation:……………………………………………….
☐ No explanation:……………………………………………….
☐ <Unknown>

Question 16. **Did it ever happen to you that you did NOT go to a doctor in the Netherlands because you were afraid that one would notice that you don't have the proper papers?**
☐ Yes explanation:……………………………………………….
☐ No explanation:……………………………………………….
☐ <Unknown>

Question 17. **Did it ever happen to you that a doctor/someone in a hospital in the Netherlands sent you away because you did not have insurance?**
☐ Yes
☐ No --> END OF INTERVIEW!! *Further with question 20.*
☐ Unknown, because person never tried --> END OF INTERVIEW!! *Further with question 20.*

Question 18. **Where was that?**
☐ Hospital, place/name…………………..
☐ General practitioner/family doctor
☐ <Unknown>

Question 19. **What kind of health problems did you have at that time?**

………………………………………….
☐ <Unknown>

Question 20. **As a last question, may I ask you from which country you originally come from?**

…………………………………….
☐ <Unknown>
